# Sulfonamide resistance in a disseminated infection caused by *Nocardia wallacei*: a case report

**DOI:** 10.1186/1752-1947-7-103

**Published:** 2013-04-11

**Authors:** Nadim Cassir, Matthieu Million, Remy Noudel, Michel Drancourt, Philippe Brouqui

**Affiliations:** 1 Unité de Recherchesur les Maladies InfectieusesTropicales et Emergentes (URMITE), UM63, CNRS 7278, IRD 198, Inserm 1095, InstitutHospitalo-UniversitaireMéditerranée-Infection, Aix-Marseille-Université, Marseille, France; 2 Service de Maladies Infectieuses et Tropicales, Hôpital Nord, Assistance Publique-Hôpitaux de Marseille, Marseille, France; 3 Service de Neurochirurgie, Hôpital Nord, Assistance Publique-Hôpitaux de Marseille, Marseille, France; 4 InstitutHospitalo-Universitaire en Maladies Infectieuses et Tropicales, Hôpital Nord, AP-HM, Chemin des Bourrelys, 13915, Marseille, cedex 20, France

**Keywords:** Neurosurgery, *Nocardia wallacei*, Sulfonamide resistance

## Abstract

**Introduction:**

Nocardial infections, although rare, are challenging for clinicians to treat. Recent contradictory reports of sulfonamide resistance have raised concerns about using this drug to treat nocardial infections.

**Case presentation:**

A 62-year-old immunocompetent Caucasian woman showed disseminated pulmonary nodules and a brain abscess by chest computed tomography and brain magnetic resonance imaging, respectively. Multidrug-resistant *Nocardia wallacei* was cultured from a stereotactic brain biopsy and confirmed by 16S ribosomal ribonucleic acid gene sequencing. After the first-line treatment failed, a long course of trimethoprim-sulfamethoxazole was prescribed with no evidence of recurrence. To the best of our knowledge, this is the first report of a *Nocardia wallacei* disseminated infection in an immunocompetent patient, and it is the first detailed description of successful treatment with trimethoprim-sulfamethoxazole despite the resistance observed *in vitro*.

**Conclusion:**

Species identification of clinical isolates is critical for diagnosis, a prediction of antimicrobial susceptibility and epidemiological tracking. In the case of *Nocardia wallacei,* the clinical outcome suggests that sulfonamides can be used for treatment despite ambiguous results from *in vitro* susceptibility tests.

## Introduction

*Nocardia* species are aerobic, Gram-positive bacteria of the order *Actinomycetales.* They are ubiquitous in the environment and live in soil, organic matter and water. They have been recognized as opportunistic human pathogens, and infection with *Nocardia* species usually occurs through inhalation or direct cutaneous inoculation. Recently, an increased number of nocardial infections, including brain abscess, have been reported in individuals without significant immunosuppression [1].

To date, more than 50 *Nocardia* species have been isolated from clinical infections, and our knowledge of new species and taxonomic relationships is constantly expanding [2]. Pijper and Pullinger reported the first instance of a *N. transvalensis* infection in 1927 after identifying it as the causative agent of a mycetoma of the foot in a South African patient [3]. In 1997, Wilson *et al.* found that the drug pattern type IV of *N. asteroides* was more closely related to *N. transvalensis* than to other members of the *N. asteroides* complex, according to their biochemical characteristics, molecular differences and antimicrobial susceptibilities [4]. In 2008, Conville *et al.* proposed that *N. asteroides* drug pattern IV be given the new species designation of *N. wallacei*[5]. To the best of our knowledge, our present report is the first case of disseminated infection caused by *N. wallacei* in an immunocompetent patient.

Recent sulfonamide susceptibility tests of *N. wallacei* isolates in different laboratories gave contradictory results, even when they were performed in duplicate [6]. This variability raises questions regarding the accurate interpretation of these susceptibility tests in clinical settings. On the one hand, there have been recent alarming reports of increased *in vitro* sulfonamide resistance in *Nocardia* species [7-9], but on the other hand, the sulfonamide antibiotic combination of trimethoprim-sulfamethoxazole (TMP-SMX) remains one of the first-choice drugs for treating nocardiosis [1]. In this case report, we present a patient who was successfully treated for a disseminated infection caused by *N. wallacei* with a long course of TMP-SMX after a first-line antibiotic therapy failure. Initial antibiotic susceptibility tests showed that the infecting organism was resistant to multiple drugs, including sulfonamides. To the best of our knowledge, this is the first case report that clearly describes the successful treatment of a disseminated infection caused by *N. wallacei* with sulfonamides despite an initial *in vitro* resistance.

## Case presentation

A 62-year-old Caucasian woman living in an urban area of Corsica was admitted to our hospital with an increasing headache, breathing failure and pleuritic chest pain on her right side. She had no relevant past medical history other than pneumonia 6 months ago. Because of the persistence of dyspnea and radiographic findings consistent with a recurrence of infection, she underwent a fibroscopic bronchoalveolar lavage 1 month before admission. The culture grew *Nocardia* species, and the susceptibility test results were pending at the time of admission. At admission, the patient was receiving a treatment of ceftriaxone (1g) and amikacin (900mg) once a day intravenously for 15 days. Her temperature was 38.2°C with 89% oxygen saturation in ambient air. Her physical and neurological examinations were unremarkable. Laboratory analyses yielded the following numbers: hemoglobin concentration, 11.8g/dL; a white blood cell count of 4 cells× 10^9^/L (79% neutrophils, 8% lymphocytes); creatinine concentration, 0.68mg/dL and moderately elevated C-reactive protein (62mg/L; normal is less than 10mg/L). Blood cultures and a human immunodeficiency virus test were all negative. Chest computed tomography (CT) revealed an interstitial distribution of multiple pulmonary nodules, fluid in the major fissure and a small pleural effusion. CT and magnetic resonance imaging (MRI) brain scans conducted upon arrival showed three nodular lesions in the left frontoparietal lobe surrounded by perilesional edema with a faint ring enhancement after contrast injection. The hyper-intense appearance of the lesions in diffusion-weighted images of the MRI scan was highly suggestive of a brain abscess. Stereotactic brain biopsy of the lesions identified a modified acid-fast variable branching filamentous bacterium that had morphology consistent with *Nocardia* species. The final *Nocardia* species culture was obtained by inoculating a Columbia agar plate containing 5% sheep blood (bioMérieux) with pus from the abscess. The culture was incubated at 37°C in a 5% carbon dioxide atmosphere. The definite identification was based on the partial sequencing of the 16S ribosomal ribonucleic acid gene (over a length of 1439 nucleotides) as previously described [10]. The sequencing results showed a 100% sequence similarity with the *N. wallacei* reference sequence (GenBank accession number GQ853074.1). No other organisms were isolated from the patient sample. The antibiotic susceptibility of the isolate was assessed on Mueller-Hinton agar (bioMérieux) using the disk method (Mast Diagnostics). The isolate was determined to be susceptible to ceftriaxone, ciprofloxacin and linezolid and resistant to amikacin, co-trimoxazole and imipenem. These results were consistent with susceptibility testing performed at the hospital of origin using the disk method. According to the recommendations of the Clinical and Laboratory Standards Institute for the susceptibility testing of *Nocardia* species, an additional antimicrobial test was performed by the microdilution method, and this test indicated that the isolate was susceptible to co-trimoxazole (minimum inhibitory concentration (MIC)<2/38mg/L) [11]. After admission, the patient received treatment with imipenem (1g/8 hours) and amikacin (900mg/24 hours) and was switched to linezolid (600mg/12 hours) and ciprofloxacin (500mg/8 hours) after receiving the susceptibility test results. Two weeks later, because of the appearance of neurological signs such as Broca’s aphasia and the extension of the cerebral abscess while on treatment, a new surgical drainage was performed by open craniotomy excision. The antibiotic regimen was switched to TMP-SMX (2400mg/day-480mg/day) and linezolid (600mg/12 hours). The linezolid was stopped after a total of 4 weeks to limit the risk of hematological toxicity. The neurological signs were improved, and the biological parameters were normalized after 1 month of treatment. Six months later, the patient presented with an isolated neutropenia and concomitant skin rash consistent with a hematological toxicity of the TMP-SMX. The treatment was then switched to moxifloxacin (400mg/24 hours, administered orally) for the remaining 6 months of the treatment period. A 1-year followup after the antibiotic regimen ceased showed no evidence of a recurrent infection.

## Discussion

Although *Nocardia* species are a rare cause of intracranial abscess and represent only 1% to 2% of all cerebral abscesses, its mortality rate (31%) is considerably greater than that of other agents that can cause cerebral abscesses (<10%) [12]. In this context, an accurate diagnosis and early treatment are challenging. Initial combination therapy with two active agents is recommended for patients with disseminated or severe nocardiosis [1]. Empirical therapy should consider the local ecology (e.g., particular strains, trends in antimicrobial susceptibility) and be adapted after the antibiotic susceptibility testing. However, these susceptibility tests may not always be reliable, as shown by the contradictory results of TMP-SMX susceptibility trends. Uhde *et al.* reported that among 765 *Nocardia* isolates submitted to the American Center for Disease Control and Prevention in Atlanta, Georgia from 1995 to 2004, 61% were resistant to SMX, and 42% were resistant to TMP-SMX [7]. In a 2011 study of 186 *Nocardia* species isolated from patients in Spain, Larruskain *et al.* reported that 16.1% were resistant to TMP-SMX [8]. A recent report from Canada stated that 43% of the 157 of *Nocardia* isolates recovered from Quebec (1988 to 2008) were resistant to TMP-SMX [9]. By contrast, other researchers have reported a low incidence of resistance to TMP-SMX. Brown-Elliot *et al.* stated that among 552 susceptibility results submitted for review, only 14 isolates (2.5%) were identified as sulfonamide-resistant, with three isolates resistant to TMP-SMX and 11 resistant to SMX [13]. These results are strikingly similar to the low incidence (2%) of sulfonamide resistance observed by Lai *et al.* in a study of *Nocardia* isolates in Taiwan [14]. The disparity among the reported data, even in the same country, is of interest because it suggests that factors other than geographic location may play a major role in the susceptibility patterns reported; the most probable factor is a difference in methodology or interpretation. The exchange of organisms between two or more accredited laboratories has been proposed as a way to enhance the accuracy of susceptibility testing [13]. Despite this recommendation, a recent multisite study by Conville *et al.* found that there was a lack of reproducibility for the sulfonamide MIC results, especially when testing *N. wallacei* and even within a particular reference laboratory [6]. Among the reasons for this variability discussed by the authors was the difficulty associated with obtaining a homogeneous organism suspension or inoculum because of the clumping ability of *Nocardia* species. Of interest, this study indicated that the disk diffusion test for sulfonamides was useful for validating the MIC results obtained by broth microdilution. In our case, the results of these two susceptibility tests were contradictory for sulfonamides.

*N. wallacei* is expected to be susceptible to TMP-SMX and resistant to amikacin [6]. Isolates reported to be resistant to sulfonamides have nonetheless been successfully treated with sulfonamides [9]. However, rare clinical reports have described the treatment failure of *Nocardia* with TMP-SMX [15]. In the present case, the clinical response to TMP-SMX was discordant with the results of the initial *in vitro* susceptibility test. Recently, large microbiological studies have raised concerns regarding the limits of interpretation of the TMX-SMX susceptibility test results. This is the first case of disseminated infection caused by *N. wallacei* that demonstrates the conflict between the *in vitro* resistance to sulfonamides and the clinical response to TMP-SMX.

## Conclusion

We consider it necessary to preserve the traditional approach to empirical therapy for infections involving *Nocardia* species, and this therapy almost always includes TMP-SMX. One must consider the inherent difficulties of interpreting MIC results when evaluating reports of resistance to TMP-SMX. In the present case, the clinical outcome and species identification guided the treatment irrespective of the results of the susceptibility test.

## Consent

Written informed consent was obtained from the patient for the publication of this case report. A copy of the written consent is available for review by the Editor-in-Chief of this journal.

## Competing interests

The authors declare that they have no competing interests.

## Authors’ contributions

NC took care of the patient and analyzed and interpreted the patient data. MM and RN took care of the patient. MD helped perform the microbiological investigations. PB took care of the patient and was a major contributor in writing the manuscript. All authors read and approved the final manuscript.
